# Dataset demonstrating the modeling of a high performance Cu(In,Ga)Se_2_ absorber based thin film photovoltaic cell

**DOI:** 10.1016/j.dib.2017.02.020

**Published:** 2017-02-12

**Authors:** Md. Asaduzzaman, Ali Newaz Bahar, Mohammad Maksudur Rahman Bhuiyan

**Affiliations:** a Department of Information and Communication Technology (ICT), Mawlana Bhashani Science and Technology University (MBSTU), Tangail 1902, Bangladesh; b University Grants Commission of Bangladesh, 29/1, Agargaon, Sher-e-Bangla Nagar, Dhaka 1207, Bangladesh

**Keywords:** Numerical modeling, CIGS, Solar cell, Efficiency, Material properties

## Abstract

The physical data of the semiconductor materials used in the design of a CIGS absorber based thin film photovoltaic cell have been presented in this data article. Besides, the values of the contact parameter and operating conditions of the cell have been reported. Furthermore, by conducting the simulation with data corresponding to the device structure: soda-lime glass (SLG) substrate/Mo back-contact/CIGS absorber/CdS buffer/intrinsic ZnO/Al-doped ZnO window/Al-grid front-contact, the solar cell performance parameters such as open circuit voltage ${(V}_{\mathrm{oc}})$, short circuit current density $J_{\mathrm{sc}}$, fill factor (FF), efficiency (η), and collection efficiency $\eta_{c}$ have been analyzed.

**Specifications Table**

**Table:** 

Subject area	*Applied physics*
More specific subject area	*Solar cell device physics*
Type of data	*Table and figure*
How data was acquired	*Numerical data for different layer materials of CIGS solar cell has been accumulated from ref*[1], [2], [3], [4], [5], [6], [7], [8], [9]*and an online simulator, ADEPT 2.1*[10]*, has been used to extract the dataset for performance parameters of the cell.*
Data format	*Filtered and analyzed*
Experimental features	*A CIGS solar cell has been structured as SLG/Mo/CIGS/CdS/i-ZnO/ZnO/Al-grid stack. Afterwards, based on the impacts of band gap, thickness, doping concentration, and others mechanical and electrical properties of the materials the values of the performance parameters have been analyzed.*
Data accessibility	*Dataset is within the data article*

**Value of the data**

• The numerical data described in Table 1 provide the properties of the constituent materials used to design a CIGS solar cell.
• Researchers could be able to use this dataset to design and analyze another theoretical model of a photovoltaic cell.
• Analyzing these data, one can compare and ensure the validity of other simulation approaches and models.
• The values of the performance parameters can be used to compare the simulation results of CIGS solar cell.

## 1 Data

The physical data for input parameters of different materials used for designing a highly efficient CIGS solar cell have been presented in Table 1. Along with this dataset, the contact parameters and the conditions under which the simulation was conducted have been demonstrated in Table 2, Table 3 respectively. All of these data has been extracted from the published literatures [1], [2], [3], [4], [5], [6], [7], [8], [9]. Fig. 2 and Table 4 describe the performance measurement parameters of the optimized CIGS absorber based photovoltaic cell.

## 2 Experimental design, materials and methods

### 2.1 Device structure of CIGS thin film photovoltaic cell

**Fig. 1:**
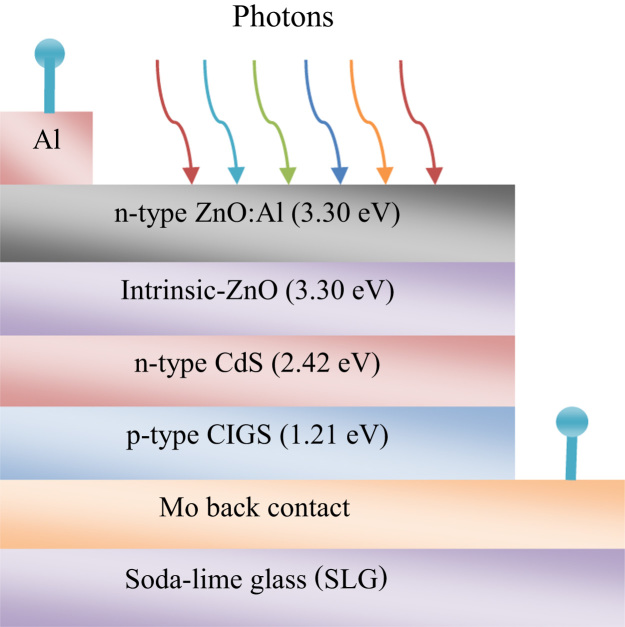
Schematic design of CIGS thin film solar cell.

The schematic design for CIGS absorber based solar cell has been depicted in Fig. 1. A soda lime glass (SLG) has been used as a substrate of the cell. After that, a stack of materials: Mo/Cu(In,Ga)Se_2_/CdS/i-ZnO/ZnO:Al/Al-grid was proposed for epitaxial growth on the substrate.

### 2.2 Performance analysis of CIGS solar cell

**Fig. 2:**
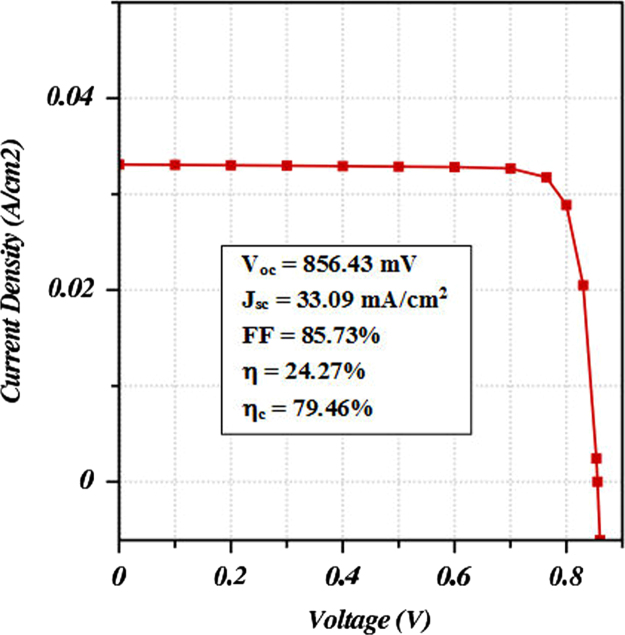
J-V characteristic curve for optimized CIGS solar cell.

ADEPT 2.1 [10], an online device simulator, has been used to simulate the design and analyze the performance of the proposed cell. The performance parameters such as $V_{\mathrm{oc}}$ and $J_{\mathrm{sc}}$ of the cell has been measured from the J-V characteristic curve as depicted in Fig. 2. Consequently, the FF, η, and $\eta_{c}$ have been determined from the simulation outcome of the cell. All of these data describing the performance of the cell are presented in Table 4.

## Figures and Tables

**Table 1:** Physical data of materials used for simulation of CIGS solar cell.

Parameters	n-ZnO: Al	i-ZnO	n-CdS	p-CIGS
Thickness, $t_{m}\left(\mathrm{µm}\right)$	0.20	0.02	0.05	3.00
Dielectric constant, $K_{s}$	7.80	7.80	8.28	13.60
Refractive index, $N_{\mathrm{dx}}$	2.00	2.00	3.16	3.67
Band gap, $E_{g}\left(\mathrm{eV}\right)$	3.30	3.30	2.42	1.21
Electron affinity, $\chi_{e}\left(\mathrm{eV}\right)$	4.60	4.60	4.40	4.21
Electron mobility, ${µ}_{n}\;({\mathrm{cm}}^{2}V^{-1}s^{-1})$	160	130	350	100
Hole mobility, ${µ}_{p}({\mathrm{cm}}^{2}V^{-1}s^{-1})$	40	30	50	25
Conduction band effective density of states, $N_{c}({\mathrm{cm}}^{-3})$	2.2×10^18^	1.5×10^18^	1.7×10^18^	2×10^18^
Valence band effective density of states, $N_{v}({\mathrm{cm}}^{-3})$	1.8×10^19^	1.6×10^19^	2.4×10^19^	1.6×10^19^
Donor concentration, $N_{d}({\mathrm{cm}}^{-3})$	1×10^18^	2×10^17^	5×10^18^	0
Acceptor concentration, $N_{a}({\mathrm{cm}}^{-3})$	0	0	0	1×10^19^
Electron lifetime, $\tau_{n}\left(s\right)$	5×10^−8^	3×10^−8^	2×10^−8^	1×10^−8^
Hole lifetime, $\tau_{p}\left(s\right)$	5×10^−9^	3×10^−9^	6×10^−8^	5×10^−8^

**Table 2:** Contact parameters for simulation of CIGS solar cell.

Parameters	Front contact	Back contact
Reflectance	0.1	0.8
Recombination velocity for holes	10^7^	10^7^
Recombination velocity for electrons	10^7^	10^7^

**Table 3:** Operating conditions based on which the simulation was carried out.

Operating conditions	Description
Illumination condition	AM1.5G
Solar irradiance on earth, $E({\mathrm{Wcm}}^{-2})$	0.1
Temperature, $T_{k}\left(K\right)$	300.15
Shadowing factor	0.05

**Table 4:** Optimized performance parameters of simulated CIGS solar cell.

Performance parameters	Parametric value
Open circuit voltage, $V_{\mathrm{oc}}\left(\mathrm{mV}\right)$	856.43
Short circuit current density, $J_{\mathrm{sc}}({\mathrm{mAcm}}^{-2})$	33.09
Fill factor, FF(%)	85.73
Efficiency, η(%)	24.27
Collection efficiency, $\eta_{c}\left(\%\right)$	79.46
